# Blood Flow of Au-Nanofluid Using Sisko Model in Stenotic Artery with Porous Walls and Viscous Dissipation Effect

**DOI:** 10.3390/mi13081303

**Published:** 2022-08-12

**Authors:** Tao-Qian Tang, Muhammad Rooman, Narcisa Vrinceanu, Zahir Shah, Ahmed Alshehri

**Affiliations:** 1 International Intercollegiate Ph.D. Program, National Tsing Hua University, Hsinchu 30013, Taiwan; 2 Department of Internal Medicine, E-Da Hospital, Kaohsiung 82445, Taiwan; 3 School of Medicine, College of Medicine, I-Shou University, Kaohsiung 82445, Taiwan; 4 Department of Family and Community Medicine, E-Da Hospital, Kaohsiung 82445, Taiwan; 5 Department of Engineering and System Science, National Tsing Hua University, Hsinchu 30013, Taiwan; 6 Department of Mathematical Sciences, University of Lakki Marwat, Lakki Marwat 28420, Pakistan; 7 Faculty of Engineering, Department of Industrial Machines and Equipments, “Lucian Blaga” University of Sibiu, 10 Victoriei Boulevard, 5500204 Sibiu, Romania; 8 Department of Mathematics, Faculty of Sciences, King Abdulaziz University, Jeddah 21589, Saudi Arabia

**Keywords:** stenosis arteries, Sisko fluid, nanofluid, blood flow, gold nanoparticles, porous medium, viscous dissipation

## Abstract

Nanofluids are extremely useful to investigators due to their greater heat transfer rates, which have significant applications in multiple industries. The primary objective of this article is to look into the effect of viscous dissipation in Sisko nano liquid flow with gold $\left(\mathrm{Au}\right)$ nanoparticles on a porous stenosis artery. Heat transfer properties were explored. Blood was utilized as a base fluid for nanoparticles. To renovate the governing nonlinear PDEs into nonlinear ODEs, appropriate transformations were used. The bvp4c-based shooting method, via MATLAB, was used to determine the numerical results of the nonlinear ODEs. Furthermore, flow forecasts for each physical quantity were explored. To demonstrate the physical influences of flow constraints versus presumed flow fields, physical explanations were used. The findings demonstrated that the velocity contour improved as the volume fraction, curvature, power law index, and material parameter upsurged. For the Prandtl number, the volume fraction of nanoparticles, the index of the power law, and the temperature profile of the nanofluid declined. Furthermore, the drag force and transfer of the heat were also investigated as explanations for influences on blood flow. Further, the Nusselt number reduced and the drag force enhanced as the curvature parameter values increased. The modeling and numerical solutions play an impressive role in predicting the cause of atherosclerosis.

## 1. Introduction

Nanoparticle properties, including surface and shape, are controlled to improve their role in a biological network. A significant amount of nanomaterials have been developed for numerous applications in biomedical science, with few of them demonstrating a tremendous ability for treating diseases or imaging. In nanomedicine and nanoscience, gold $\left(\mathrm{Au}\right)$ nanoparticles are the most potent materials. In fact, small gold nanoparticles are used in a variety of biomedical applications, such as stimulating blood vessel growth. They are also used to transport drugs. The effects of arterial stenosis on blood (non-Newtonian fluid) with Au nanoparticles are discussed in this article. Plaques or narrowings in the human arterial system, identified as arterial stenosis, are quite common. The stenosis disrupts the usual pattern of blood flow over the artery. To filter waste products, our kidneys require a certain amount of blood flow. As a result of artery narrowings, our kidneys cannot attain normal amounts of oxygen-rich blood, resulting in numerous injuries and an escalation in blood pressure. Ellahi et al. [1] used the perturbation method to solve the problem of two-dimensional (2D) blood flows over porous stenosis walls. Ardahaie et al. [2] numerically and analytically examined the blood motion (using a third-grade model) with the nanoparticle concentration on absorbent surfaces. Haghighi et al. [3] characterized a 2D blood flow mathematical model across tapered blood vessels and used FDM to simplify the model. Kanai et al. [4] analytically demonstrated the need for an appropriately sized catheter for each experiment to minimize inaccuracies due to wave reflection at the catheter tip. Leimgruber et al. [5] observed a large mean pressure gradient through the stenosis throughout the balloon angioplasty. Back et al. [6] and Back [7] scrutinized the boost in mean flow opposition during normal and stenosed coronary artery catheterization. Sarkar and Jayaraman [8] addressed how pulsatile blood flow patterns altered in a stenosed catheterized artery. Dash et al. [9] also investigated the issue in a curved stenosed artery. Recently, Sarwar and Hussain [10] explored human blood flow behavior under stenosis presumptions.

One of the prominent issues in the last few decades has been the exploration of non-Newtonian fluids regarding extending surfaces. These fluids have attracted attention because of their numerous modern and unique uses, such as ink-jet printing, polymer handling, geographical streams, etc. Sisko fluid is a non-Newtonian fluid model that is, comparatively, simple and straightforward. It is essentially an extension of the Ostwald de Waele model formulated by Sisko [11] in 1958. The Newtonian and non-Newtonian fluids are combined in the Sisko fluid model. Such fluids are common in nature and have a wide range of new applications; the flow of lubricating oils is a prime example of this type of fluid. Khan et al. [12] recently inspected the geometry of an annular pipe and measured the computational and analytic solutions of 2D, steady flow, as well as the heat transfer characteristics, of the Sisko fluid. They discovered that the velocity of viscous fluids was much lower than that of the Sisko fluid. They discovered that boosting the flow behavior index strengthens the strong shear thickening influence. Nadeem et al. [13] investigated the qualities of Sisko fluid peristaltic pumping in a consistent tube. They tested the liquid model for distinct flow behavior quantiles and unearthed that viscous fluid has the ideal peristaltic pumping qualities. Munir et al. [14] evaluated the floatation consequences of Sisko fluid over a surface that is isothermally stretched, both favorably and unfavorably. Hayat et al. [15] used the Sisko fluid model to simulate flow in an absorbent medium. Sari et al. [16] explored the dynamic flow of Sisko liquid close to a stagnation point using the Lie group theory.

The utilization of nanofluid as an effective cooling channel in nuclear power plants signifies that the operating coolant can be utilized to cool heated surfaces more efficiently. In nanofluid uses, a heat valve is utilized to handle the heat flow. Their ability to quickly transfer heat could be used to cool down research objects. Medical oncologists treat cancer sufferers by injecting medications and radiation into a computer made of nanofluid with iron as the foundation. Because of the countless usages of the latest innovation’s transfer of heat and energy cycles, nanotechnology has, in recent times, piqued the interest of experts. It has significantly advanced heat transfer science by designing new liquids, known as nanofluids, which reduce the size of heat transfer gear while improving energy efficiency. Choi and Eastman [17] investigated the nanostructure’s possibility of diffraction in the base fluid. Hatami et al. [18] inspected the transfer of the nanofluid heat properties in leaky media using multiple analytic techniques. Akbari et al. [19] explored magnetized transference of the third-degree, blood-based Au nanoparticles over porous arteries using Flex PDE programming. Srinivas et al. [20] reviewed the rate of heat transport of Au nanoparticles in blood on the surface medium. Hady et al. [21] looked into the transfer of heat properties of nanoparticles in permeable media. As stated by Buongiorno [22], nanofluids are more stable than ordinary liquids and have better spreading, wetting, and scattering functionality around the outer layer of solids. Hady et al. [23] studied nanofluid flow in the existence of yield stress. In this case, the surface is dispersed nonlinearly. In the existence of first-order chemical reactions, thermal radiations, stagnation points, and heat absorption/generation impacts, Khalil et al. [24] evaluated the transfer of heat through a double sampling of stratification in non-Newtonian magnetized fluid flow to inclined stretched surfaces. They used the shooting method, along with Runge–Kutta methods, to find the numerical solution. The most recent essential outcomes on non-Newtonian flow in numerous patterns can be found in references [25,26,27,28,29].

The current study’s purpose was to investigate the Sisko nanofluid flow containing Au NPs through porous stenosed arteries. Blood was used as a base fluid for nanoparticles. The heat transfer properties with viscous dissipation were investigated. The influences of several parameters, such as the volume fraction, the Prandtl number, and the blood flow parameter, are discussed, and the findings are reported in figures and tables. The article is organized as follows: first, the governing equations are tackled, and then a numerical result is acquired using Shooting Scheme bvp4c via MATLAB software. Second, the physical quantities of various parameters are explained by plotting figures. Finally, closing remarks are provided. The present investigation is significant for a variety of biomedical applications.

## 2. Mathematical Formulation

We assumed, in the present issue, that blood behaves as a steady, incompressible, porous, non-Newtonian Sisko fluid flow via the length of an arterial stenosis $\;\frac{L_{0}}{2}$. The coordinate system was chosen so that blood flow along the x-axis and r-axis could be chosen to take perpendicular blood flow. In the problem’s schematic diagram, blood flows through an artery to a cosine shape stenosis with an unimpeded width, where $2R_{0}$, $R\left(x\right)$ is the radius of the artery and λ is the highest possible height of the stenosis. The following profile was selected for the stenosed region:

(1) $$R\left(x\right)=R_{0}-\frac{\lambda }{2}\left(1+Cos\left(\frac{4\pi x}{L_{0}}\right)\right),\;-\frac{L_{0}}{4}<x<\frac{L_{0}}{4}=R_{0}\;Otherwise$$

With these suppositions, the steady boundary layer equations governing the flow and heat transfer of non-Newtonian Sisko nanofluid were defined as follows [10,29]:

(2) $$\frac{\partial \left(ru\right)}{\partial x}+\frac{\partial \left(rv\right)}{\partial r}=0$$

(3) $$\rho_{nf}\left(u\frac{\partial u}{\partial x}+v\frac{\partial u}{\partial r}\right)=\frac{\mu_{nf}}{r}\frac{\partial }{\partial r}\left(r\frac{\partial u}{\partial r}\right)-\frac{\alpha }{r}\frac{\partial }{\partial r}\left(r{\left(-\frac{\partial u}{\partial r}\right)}^{n}\right)-\frac{\mu_{nf}}{K}u$$

(4) $${\left(\rho C_{p}\right)}_{nf}\left(u\frac{\partial T}{\partial x}+v\frac{\partial T}{\partial r}\right)=\frac{k_{nf}}{r}\frac{\partial }{\partial r}\left(r\frac{\partial u}{\partial r}\right)+\mu_{nf}{\left(-\frac{\partial u}{\partial r}\right)}^{2}+\alpha {\left(-\frac{\partial u}{\partial r}\right)}^{n+1}$$

as well as boundary conditions:

(5) $$\begin{array}{c} u=u_{0},\;\;v=0,\;\;T=T_{w}\;\;at\;\;r=R\\ u\to 0,\;\;T\to T_{\infty }\;\;\;at\;\;\;r\to \infty \end{array}$$

where $\mu_{nf},\;\rho_{nf},\;{\left(\rho C_{p}\right)}_{nf}\;,and\;\;k_{nf}$ were defined as [10]. The physical properties of base fluid (blood) and nanoparticle are given in Table 1.

(6) $$\left.\begin{array}{c} \begin{array}{c} \mu_{nf}=\mu_{f}{\left(1-\phi \right)}^{-2.5}\\ \rho_{nf}=\rho_{f}\left(1-\phi \right)+\phi \rho_{s}\\ {\left(\rho c_{p}\right)}_{nf}={\left(\rho c_{p}\right)}_{f}\left(1-\phi \right)+\phi {\left(\rho c_{p}\right)}_{s} \end{array}\\ k_{nf}=k_{f}\left[\frac{k_{s}+2k_{f}-2\phi \left(k_{f}-k_{s}\right)}{k_{s}+2k_{f}+2\phi \left(k_{f}-k_{s}\right)}\right] \end{array}\right\}$$

We took into consideration the following transformation:

(7) $$u=\frac{u_{0}x}{L_{0}}F^{\prime }\left(\eta \right),\;v=-\frac{R}{r}\sqrt{\frac{u_{0}\upsilon_{f}}{L_{0}}}F\left(\eta \right),\;\theta \left(\eta \right)=\frac{T-T_{\infty }}{T_{w}-T_{\infty }},\;\eta =\frac{r^{2}-R^{2}}{2R}\sqrt{\frac{u_{0}}{\upsilon_{f}L_{0}}}$$

After implementing the similarity transformation, the continuity Equation (2) was satisfied and Equations (3) and (4) took the following form:

(8) $$\begin{array}{c} \left(1+2\eta \gamma \right)F^{\prime \prime \prime }+2\gamma F^{\prime \prime }+\varphi_{1}S{\left(1+2\eta \gamma \right)}^{\frac{n-1}{2}}\left(n\left(1+2\eta \gamma \right){\left(-F^{\prime \prime }\right)}^{n-1}F^{\prime \prime \prime }-\gamma {\left(-F^{\prime \prime }\right)}^{n}\right)\\ -\beta F^{\prime }-\varphi_{1}\varphi_{2}\left({F^{\prime }}^{2}-FF^{\prime \prime }\right)=0 \end{array}$$

(9) $$\begin{array}{c} \varphi_{4}\left(\left(1+2\eta \gamma \right)\theta^{\prime \prime }+2\gamma \theta^{\prime }\right)+\varphi_{3}PrF\theta^{\prime }\\ +PrEc\left(\frac{\left(1+2\eta \gamma \right)}{\varphi_{1}}{F^{\prime \prime }}^{2}+S{\left(1+2\eta \gamma \right)}^{\frac{n+1}{2}}{\left(-F^{\prime \prime }\right)}^{n}\right)=0 \end{array}$$

Nondimensional boundary conditions were defined as:

(10) $$\begin{array}{c} F\left(0\right)=0,\;\;F^{\prime }\left(0\right)=1,\;\;\theta \left(0\right)=1\\ F^{\prime }\left(\eta \right)\to 0,\;\;\theta \left(\eta \right)\to 0,\;\;as\;\;\eta \to \infty \end{array}$$

In Equations (8) and (9), the dimensionless parameters were:

(11) $$S=\frac{\alpha }{\mu_{f}}{\left(\frac{u_{0}x}{L_{0}}\sqrt{\frac{u_{0}}{\upsilon_{f}L_{0}}}\right)}^{n-1},\;\gamma =\sqrt{\frac{\upsilon_{f}L_{0}}{u_{0}R^{2}}},\;\beta =\frac{\mu_{f}L_{0}}{\rho_{f}u_{0}K},\;Pr=\frac{{\left(\rho C_{p}\right)}_{nf}\upsilon_{f}}{k_{f}},\;Ec=\frac{u_{0}{}^{2}x^{2}}{C_{p}\left(T_{w}-T_{\infty }\right)L_{0}{}^{2}}$$

Also, we take

(12) $$\begin{array}{c} \varphi_{1}={\left(1-\phi \right)}^{2.5},\;\varphi_{2}=\left(1-\phi \right)+\phi \frac{\rho_{s}}{\rho_{f}},\;\varphi_{3}=\left(1-\phi \right)+\phi \frac{{\left(\rho C_{p}\right)}_{s}}{{\left(\rho C_{p}\right)}_{f}},\\ \varphi_{4}=\frac{k_{s}+2k_{f}-2\phi \left(k_{f}-k_{s}\right)}{k_{s}+2k_{f}+2\phi \left(k_{f}-k_{s}\right)} \end{array}$$

The physical quantities of the flow field, namely the skin friction coefficient $C_{f}$ and the heat transfer coefficient $Nu_{x}$, were classified as follows:

(13) $$\begin{array}{c} C_{f}=\frac{2\tau_{w}}{\rho_{f}u_{w}{}^{2}}\\ Nu=\frac{xq_{w}}{k_{f}\left(T_{w}-T_{\infty }\right)} \end{array}$$

The shear stress $\tau_{w}$ and heat flux $q_{w}$ was defined as:

(14) $$\begin{array}{c} \tau_{w}={\left.\left[\mu_{nf}\frac{\partial u}{\partial r}-\alpha {\left(-\frac{\partial u}{\partial r}\right)}^{n}\right]\right|}_{r=R}\\ q_{w}=-k_{nf}{\left.\frac{\partial T}{\partial r}\right|}_{r=R} \end{array}$$

The non-dimensional forms of Equations (13) and (14) were:

(15) $$\begin{array}{c} Re_{x}{}^{1/2}C_{f}=\frac{1}{\varphi_{1}}F^{\prime \prime }\left(0\right)-S{\left(-F^{\prime \prime }\left(0\right)\right)}^{n}\\ Re_{x}{}^{-1/2}Nu_{x}=-\frac{k_{nf}}{k_{f}}\theta^{\prime }\left(0\right) \end{array}$$

## 3. Numerical Method

This section computes the solution framework for the constructed model using the bvp4c method (shooting scheme). The MATLAB tool’s bvp4c method (shooting scheme) was employed to numerically solve the ODEs (8)–(9) via (10). For this methodology, we first converted a higher-order system into a first-order system. We took the steps listed below to accomplish this:

(16) $$\begin{array}{c} F=z_{1},\;F^{\prime }=z_{2},\;F^{\prime \prime }=z_{3},\;F^{\prime \prime \prime }=z_{3}^{\prime }\\ \theta =z_{4},\;\theta^{\prime }=z_{5},\;\theta^{\prime \prime }=z_{5}^{\prime } \end{array}$$

(17) $$z_{3}^{\prime }=\frac{\varphi_{1}S{\left(1+2\eta \gamma \right)}^{\frac{n-1}{2}}\gamma {\left(-z_{3}\right)}^{n}-2\gamma z_{3}+\beta z_{2}+\varphi_{1}\varphi_{2}\left(z_{2}{}^{2}-z_{1}z_{3}\right)}{\left(1+2\eta \gamma \right)\left(1+\varphi_{1}Sn{\left(1+2\eta \gamma \right)}^{\frac{n-1}{2}}{\left(-z_{3}\right)}^{n-1}\right)}$$

(18) $$\begin{array}{cc} z_{5}^{\prime }=\frac{1}{\left(1+2\eta \gamma \right)} & [-2\gamma z_{5}\\ & -\frac{1}{\varphi_{4}}(\varphi_{3}Prz_{1}z_{5}\\ & +PrEc(\frac{\left(1+2\eta \gamma \right)}{\varphi_{1}}z_{3}{}^{2}+S{\left(1+2\eta \gamma \right)}^{\frac{n+1}{2}}{\left(-z_{3}\right)}^{n}))] \end{array}$$

with boundary conditions:

(19) $$\begin{array}{c} z_{1}\left(0\right)=0,\;z_{2}\left(0\right)=1,\;z_{4}\left(0\right)=1\\ z_{2}\left(\eta \right)\to 0,\;z_{4}\left(\eta \right)\to 0,\;as\;\eta \to \infty \end{array}$$

## 4. Results and Discussion

**Figure 1:**
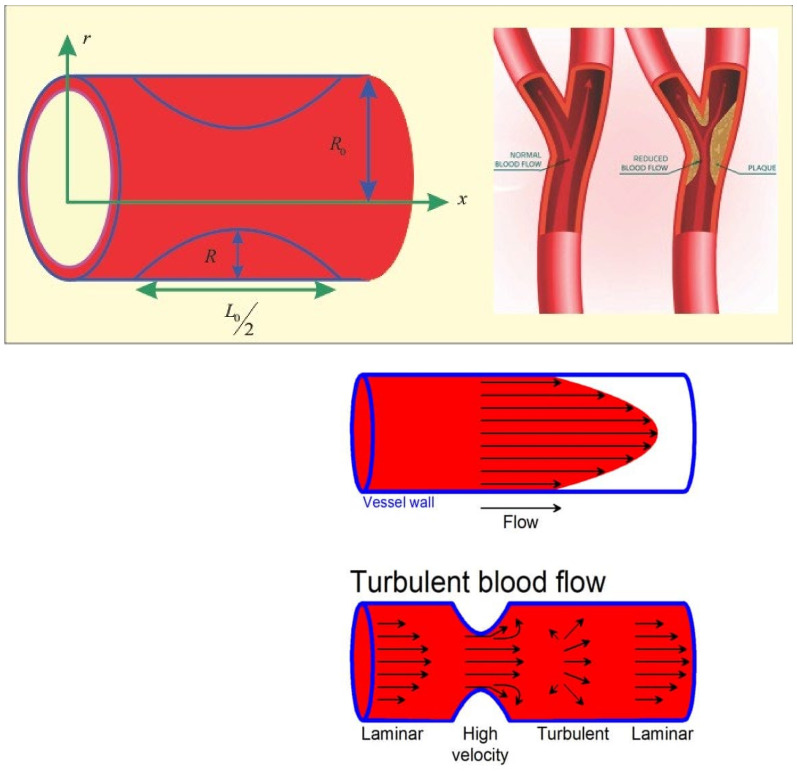
Geometry of the problem.

**Figure 2:**
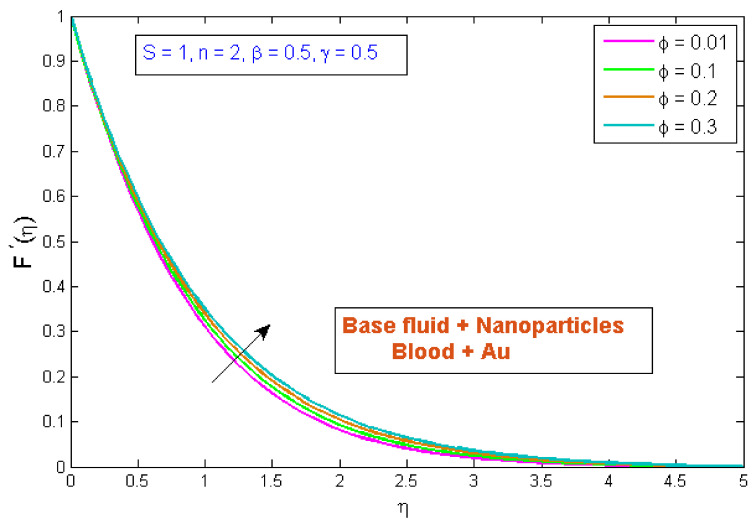
Modification of $F^{\prime }\left(\eta \right)$ versus ϕ.

**Figure 3:**
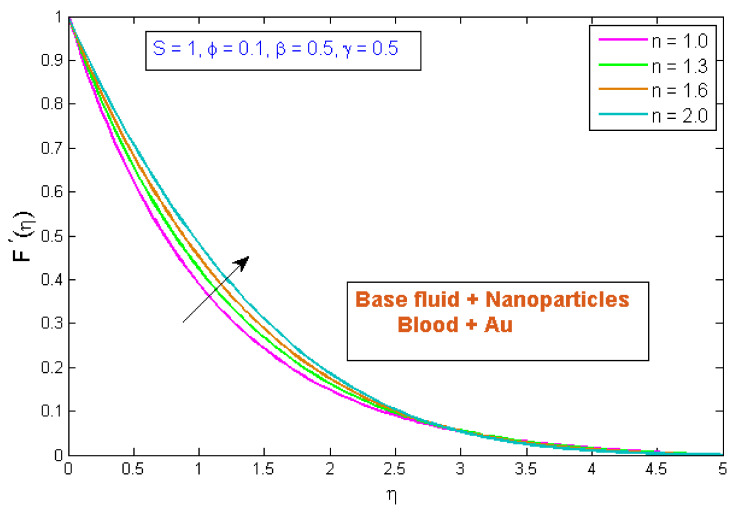
Modification of $F^{\prime }\left(\eta \right)$ versus n.

**Figure 4:**
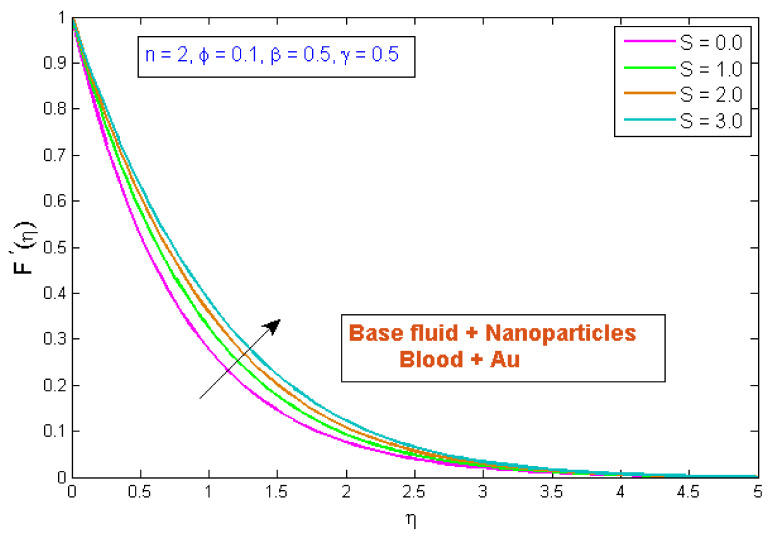
Modification of $F^{\prime }\left(\eta \right)$ versus S.

**Figure 5:**
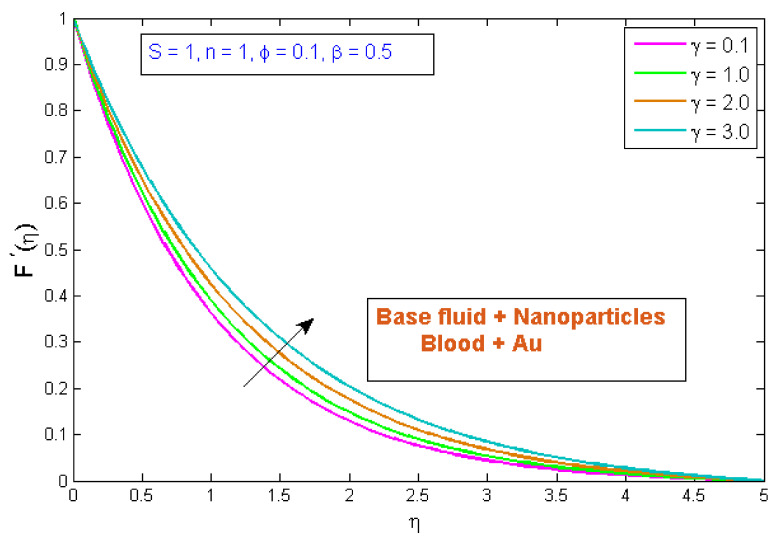
Modification of $F^{\prime }\left(\eta \right)$ versus γ.

**Figure 6:**
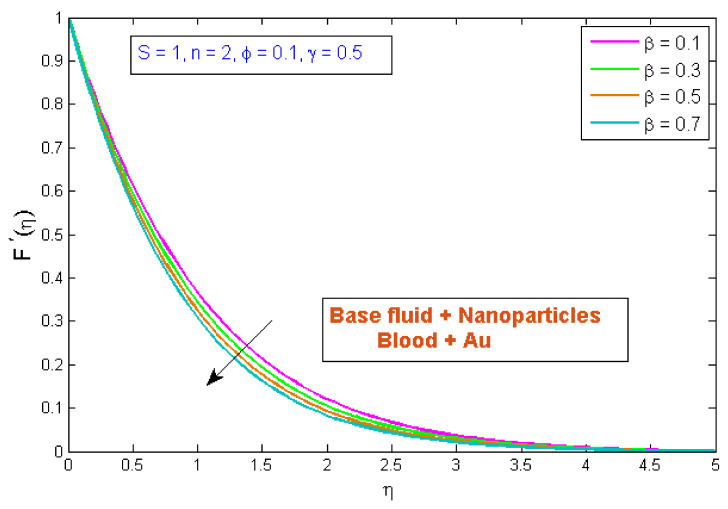
Modification of $F^{\prime }\left(\eta \right)$ versus β.

**Figure 7:**
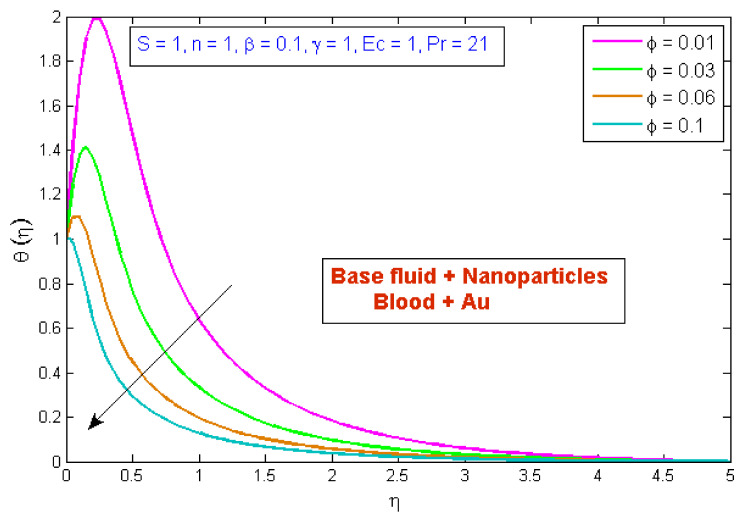
Modification of $\theta \left(\eta \right)$ versus ϕ.

**Figure 8:**
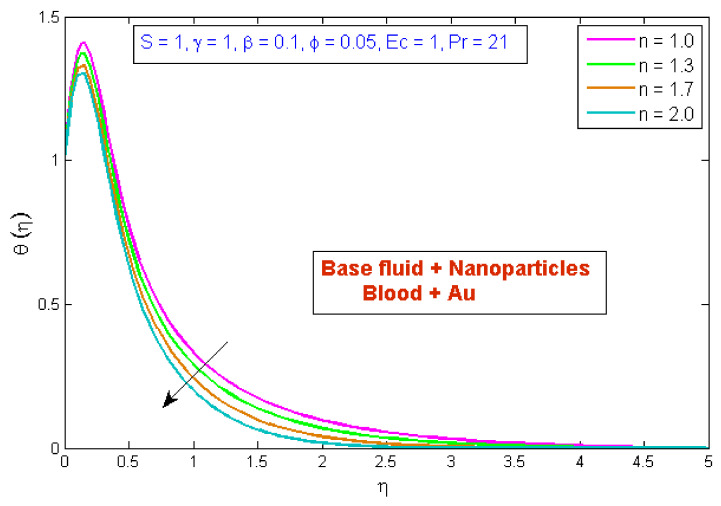
Modification of $\theta \left(\eta \right)$ versus n.

**Figure 9:**
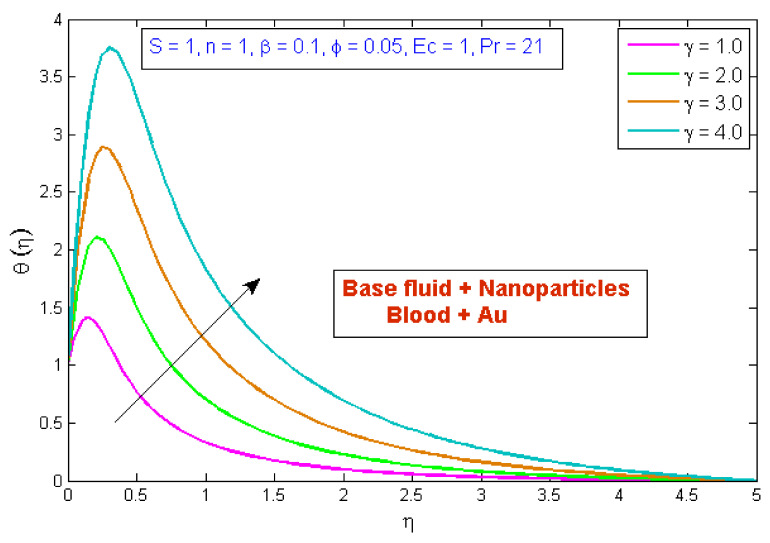
Modification of $\theta \left(\eta \right)$ versus γ.

**Figure 10:**
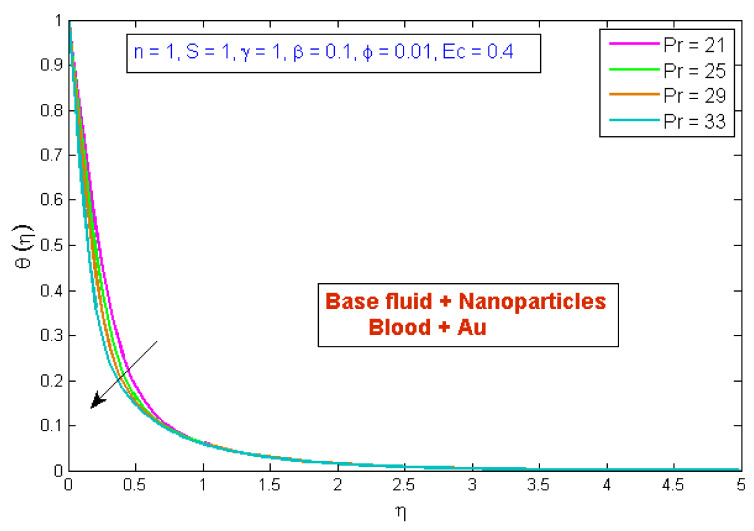
Modification of $\theta \left(\eta \right)$ versus Pr.

**Figure 11:**
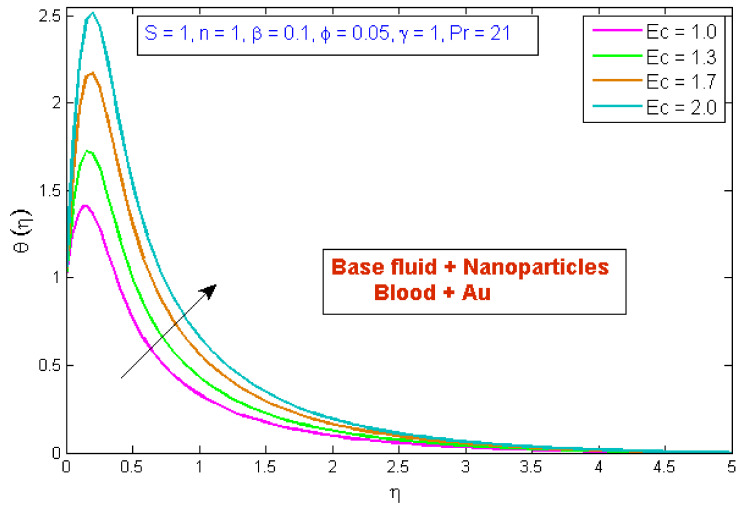
Modification of $\theta \left(\eta \right)$ versus Ec.

This section explains the effects of various parameters in great detail. Normal blood vessels and blood vessels with plaque are depicted in the graphical abstract. The geometrical structure of an artery is depicted in Figure 1. Gold $\left(\mathrm{Au}\right)$ nanoparticles and blood as the base fluid were used to measure the flow and transfer of heat qualities of stenosed arteries. The influences of relating flow constraints on the temperature and velocity fields were physically examined and displayed. Figure 2, Figure 3, Figure 4, Figure 5, Figure 6, Figure 7, Figure 8, Figure 9, Figure 10 and Figure 11 have been mapped for this purpose. To obtain numerical solutions for the ODEs, the bvp4c (shooting) technique was utilized.

### 4.1. Velocity Profile

Figure 2 indicates the influence of the volume fraction of gold nanoparticles, ϕ, on the velocity profile, $F^{\prime }\left(\eta \right)$. The variation was checked on four different values of ϕ, while other parameters were fixed. The outcomes indicated that the velocity profile improved as the gold nanoparticles’ volume fraction increased, implying that utilizing gold nanoparticles can strengthen blood velocity in stenosed arteries. Furthermore, the blood velocity has been shown to be rapid in the center of the vessel and least efficient at the vessel wall. Figure 3 demonstrates the inspiration of the power law index, n, on the velocity profile, $F^{\prime }\left(\eta \right)$. It has been perceived that an uptick in the power law index, n, causes a growth in velocity. The physical cause for this phenomenon is that shear-thinning fluid encounters less resistance, owing to its poor viscosity, and leads to a rise in the fluid. Figure 4 depicts the behavior of the velocity profile, $F^{\prime }\left(\eta \right)$, for different Sisko fluid material parameter values, S. It was demonstrated that raising the fluid parameter, S, improved the velocity profile. S is described as the viscosity of a high rate of shear to the index of the consistency ratio. Since raising S improves the initial forces of the fluid, resulting in a decrement in viscous forces, a boost in velocity occurs. Figure 5 displays the stimulus of curvature parameter, γ, on velocity, $F^{\prime }\left(\eta \right)$. This figure shows that the velocity improved with rises in the curvature parameter. It has been determined that as the curvature parameter rises, the velocity field improves because the radius of the curvature declines, thus leading to a reduction in the contact area between the fluid and the cylinder. As a result, the cylinder’s surface offers less resistance to fluid motion. Figure 6 depicts the consequences of the porosity parameter, β, on the blood flow velocity profile. The figure shows that as the porosity parameter was increased, the velocity profile diminished. As a result, for a given value of β, the velocity reaches an extreme point in the middle of the artery and begins to decrease near the artery’s wall. The velocity profile with the β demonstrated this behavior, which may be due to the fact that as the fraction of void volume over total volume is boosted, fluid movement in the artery becomes incredibly hard. As a result, as β improved, the velocity through a porous stenosed artery was reduced. 

### 4.2. Temperature Profile

Figure 7 depicts the effect of the volume fraction of gold nanoparticles on the temperature profile, $\theta \left(\eta \right)$. The graph shows that boosting the nanofluid volume fraction, ϕ, reduced temperature. This reduction is due to gold nanoparticles’ high thermal conductivity, which plays a significant role in rapidly dissipating temperature. Figure 8 demonstrates the stimulus of the power law index, n, on the temperature profile, $\theta \left(\eta \right)$. The physical cause for this phenomenon is that shear-thinning fluid encounters less resistance owing to its poor viscosity, thus resulting in augmented fluid velocity and diminished fluid temperature. Figure 9 describes the consequence of the curvature parameter, γ, on temperature distribution, revealing that temperature demonstrates an inciting trend via γ. An uptick in γ diminishes the surface link area of the liquid particles, resulting in less opposition and an upswing in velocity. Since the Kelvin temperature is described by average kinetic energy, the temperature rises. Figure 10 depicts the effect of the temperature profile, $\theta \left(\eta \right)$, versus the Prandtl number, Pr. It can be seen that as Pr upturned, the temperature of the fluid decreased. This is because the thermal boundary thickness was reduced as the Pr grew. This signifies that an uptick in Pr is related to an upsurge in the heat transfer rate at the blood arterial wall. When blood has an advanced Pr, its thermal conductivity declines; thus, its capacity for heat conduction is significantly reduced, and the fluid temperature is reduced. Figure 11 portrays the disparity in fluid temperature caused by fluctuations in the Eckert number, Ec. The graph shows that as the value of Ec upswung, so did the fluid temperature. Because of frictional heating, heat was created in the fluid as the value of Ec upsurged. Ec is usually physically defined as the ratio of kinetic energy to the specific enthalpy variance between the wall and the fluid. Consequently, an upsurge in the Ec findings in the conversion of kinetic energy into internal energy via work was conducted against viscous fluid stresses. Therefore, raising the Ec resulted in an upsurge of the fluid temperature.

## 5. Physical Quantities

The effects of the gold nanoparticles’ volume fraction, Sisko fluid parameter, curvature parameter, and porosity parameter on the skin friction coefficient are portrayed in Table 2. The skin friction coefficient declined as the numbers for the volume friction, Sisko fluid parameter, and porosity parameter increased, whereas the skin friction coefficient rose as the curvature parameter was enhanced. Table 3 shows how the Nusselt number changed as the physical parameters’ volume fractions, curvature parameters, and Eckert numbers changed. The Nusselt number grows when large values are awarded to volume fraction rises. On the other hand, as the values of the physical parameters Ec and γ boost, the local Nusselt number declines. The findings were compared to existing data and were found to be in satisfactory agreement, as shown in Table 4.

## 6. Conclusions

The primary objective of this research was to look into the effect of Sisko nanofluid flow with Au nanoparticles on a porous stenosed artery. The heat transfer properties, together with viscous dissipation, were investigated. Blood was used as a base fluid for nanoparticles. To renovate the governing PDEs into nonlinear ODEs, appropriate transformations were used. The shooting method (bvp4c) via MATLAB was used to determine the numerical solutions for the nonlinear ODEs. The following are the main findings of the present study:

- When the volume fraction, fluid parameter, power law index, and curvature parameter were increased, the velocity profile decreased.
- When the porosity parameter was augmented, the velocity profile was diminished.
- The temperature profile was reduced as the volume fraction, power law index, and Prandtl number values were increased.
- The temperature profile was boosted as the curvature parameter and the Eckert number upsurged.
- The skin friction coefficient declined as the volume friction, Sisko fluid parameter, and porosity parameter increased, while the skin friction coefficient rose as the curvature parameter was enhanced.
- The Nusselt number grew when large value increases were attained in the volume fraction. Instead, as the values of the physical parameters Ec and γ were boosted, the local Nusselt number declined.

## Figures and Tables

**Table 1:** Base fluid (blood) and nanoparticle experimental values [10].

Material	Symbol	$\rho \left(kg/m^{3}\right)$	$C_{p}\left(J/kg\;K\right)$	$k\;\;\left(W/m\;K\right)$
**Blood**	--	1050	3617	0.52
**Gold**	Au	19,300	129	318

**Table 2:** Skin friction coefficient variations for various parameters.

ϕ	S	γ	β	$Re_{x}{}^{1/2}C_{f}$
0.01	0.1	0.1	0.1	−1.053197
0.05				−1.094823
0.1				−1.155339
0.01	0.1			−1.053197
	0.3			−1.16986
	0.5			−1.278185
	0.1	0.1		−1.053197
		0.2		−0.9924828
		0.3		−0.9448766
		0.1	0.1	−1.053197
			0.3	−1.138746
			0.5	−1.218283

**Table 3:** Nusselt number variations for various parameters.

ϕ	γ	Ec	$Re_{x}{}^{-1/2}Nu_{x}$
0.01	0.1	0.1	3.928399
0.05			7.487721
0.1			10.59055
0.01	0.1		3.928399
	0.2		3.744353
	0.3		3.573028
	0.1	0.1	3.928399
		0.2	3.183704
		0.3	2.43899

**Table 4:** Comparisons with a previous study, when S=0,  β=0.

γ	ϕ	Present Paper $Re_{x}{}^{1/2}C_{f}$	L. Sarwar and A. Hussain [10] $Re_{x}{}^{1/2}C_{f}$
0.1	0.0	−0.93947	−0.939968
0.12		−0.9295648	−0.924794
0.14		−0.9180573	−0.911311
0.1	0.0	−0.93947	−0.939968
	0.05	−1.323752	−1.329552
	0.1	−1.714007	−1.715985

## Nomenclature

$C_{p}$	Specific heat transfer $\left(J\;/\mathrm{kg}/K\right)$
Ec	Eckert number
k	Thermal conduction $\left({W\;m}^{-1}{\;K}^{-1}\right)$
K	Porousness medium
n	Power law index
Pr	Prandtl number
S	Dimensionless Fluid parameter
T	Temperature
u, v	Components of velocity $\left({m\;s}^{-1}\right)$
r, θ, x	Coordinates axis $\left(m\right)$
**Greek Letter**	
α	Fluid parameter
β	Porosity parameter
γ	Curvature parameter
ϕ	volume fraction parameter
η	Independent coordinate
μ	Dynamic viscosity
υ	Kinematic viscosity
ρ	Density $\left(\mathrm{kg}\;m^{-3}\right)$
$\rho C_{p}$	Heat capacitance
**Subscripts**	
f	Base fluid
s	Solid nanoparticle
nf	Nanoparticle
w	Wall
∞	Free-Stream

## References

[1] Ellahi R., Rahman S.U., Nadeem S., Vafai K. (2015). The blood flow of prandtl fluid through a tapered stenosed arteries in permeable walls with magnetic field. Commun. Theor. Phys..

[2] Ardahaie S.S., Amiri A.J., Amouei A., Hosseinzadeh K., Ganji D.D. (2018). Investigating the effect of adding nanoparticles to the blood flow in presence of magnetic field in a porous blood arterial. Inform. Med. Unlocked.

[3] Haghighi A.R., Asl M.S. (2015). Mathematical modeling of micropolar fluid flow through an overlapping arterial stenosis. Int. J. Biomath..

[4] Kanai H., Iizuka M., Sakamoto K. (1970). One of the problems in the measurement of blood pressure by catheter-insertion: Wave reflection at the tip of the catheter. Med. Biol. Eng..

[5] Leimgruber P.P., Roubin G.S., Anderson H.V., Bredlau C.E., Whitworth H.B., Douglas J.S., King S.B., Greuntzig A.R. (1985). Influence of intimal dissection on restenosis after successful coronary angioplasty. Circulation.

[6] Back L.H., Kwack E.Y., Back M.R. (1996). Flow rate-pressure drop relation in coronary angioplasty: Catheter obstruction effect. J. Biomech. Eng..

[7] Back L.H. (1994). Estimated mean flow resistance increase during coronary artery catheterization. J. Biomech..

[8] Sarkar A., Jayaraman G. (1998). Correction to flow rate—Pressure drop relation in coronary angioplasty: Steady streaming effect. J. Biomech..

[9] Dash R.K., Jayaraman G., Mehta K.N. (1999). Flow in a catheterized curved artery with stenosis. J. Biomech..

[10] Sarwar L., Hussain A. (2021). Flow characteristics of Au-blood nanofluid in stenotic artery. Int. Commun. Heat Mass Transf..

[11] Sisko A.W. (2002). The flow of lubricating greases. Ind. Eng. Chem..

[12] Khan M., Munawar S., Abbasbandy S. (2010). Steady flow and heat transfer of a sisko fluid in annular pipe. Int. J. Heat Mass Transf..

[13] Nadeem S., Akbar N.S. (2010). Peristaltic flow of sisko fluid in a uniform inclined tube. Acta Mech. Sin..

[14] Munir A., Shahzad A., Khan M. (2015). Mixed convection heat transfer in sisko fluid with viscous dissipation: Effects of assisting and opposing buoyancy. Chem. Eng. Res. Des..

[15] Hayat T., Moitsheki R.J., Abelman S. (2010). Stokes’ first problem for sisko fluid over a porous wall. Appl. Math. Comput..

[16] Sari G., Pakdemirli M., Hayat T., Aksoy Y. (2012). Boundary layer equations and lie group analysis of a sisko fluid. J. Appl. Math..

[17] Choi S.U.S., Eastman J.A. Enhancing thermal conductivity of fluids with nanoparticles. Proceedings of the 1995 International Mechanical Engineering Congress and Exhibition.

[18] Hatami M., Hatami J., Ganji D.D. (2014). Computer simulation of MHD blood conveying gold nanoparticles as a third grade non-Newtonian nanofluid in a hollow porous vessel. Comput. Methods Programs Biomed..

[19] Akbari N., Ganji D.D., Gholinia M., Gholinia S. (2017). Computer simulation of blood flow with nanoparticles in a magnetic field as a third grade non-Newtonian through porous vessels by flex PDE software. Innov. Energy Res..

[20] Srinivas S., Vijayalakshmi A., Subramanyam Reddy A. (2016). Flow and heat transfer of gold-blood nanofluid in a porous channel with moving/stationary walls. J. Mech..

[21] Hady F.M., Ibrahim F.S., Abdel-Gaied S.M., Eid M.R. (2012). Radiation effect on viscous flow of a nanofluid and heat transfer over a nonlinearly stretching sheet. Nanoscale Res. Lett..

[22] Buongiorno J. (2006). Convective transport in nanofluids. J. Heat Transf..

[23] Hady F., Eid M.R., Abdelhafez M. (2014). A nanofluid flow in a non-linear stretching surface saturated in a porous medium with yield stress effect. Appl. Math. Inf. Sci. Lett..

[24] Rehman K.U., Shatanawi W., Malik M.Y. (2022). Heat transfer and double sampling of stratification phenomena in non-Newtonian liquid suspension: A comparative thermal analysis. Case Stud. Therm. Eng..

[25] Rehman K.U., Shatanawi W., Ashraf S., Kousar N. (2022). Numerical analysis of Newtonian heating convective flow by way of two different surfaces. Appl. Sci..

[26] Rehman K.U., Shatanawi W., Abodayeh K., Shatanawi T.A.M. (2022). A group theoretic analysis of mutual interactions of heat and mass transfer in a thermally slip semi-infinite domain. Appl. Sci..

[27] Dawar A., Islam S., Tassaddiq A., Shah Z., Deebani W., Rashid A. (2022). Mixed convective flow of blood biofluids containing magnetite ferroparticles past a vertical flat plate: Shapes-based analysis. Waves Random Complex Media.

[28] Alsagri A.S., Nasir S., Gul T., Islam S., Nisar K.S., Shah Z., Khan I. (2019). MHD thin film flow and thermal analysis of blood with CNTs nanofluid. Coatings.

[29] Awais M., Malik M.Y., Bilal S., Salahuddin T., Hussain A. (2017). Magnetohydrodynamic (MHD) flow of sisko fluid near the axisymmetric stagnation point towards a stretching cylinder. Results Phys..

